# BioVR: a platform for virtual reality assisted biological data integration and visualization

**DOI:** 10.1186/s12859-019-2666-z

**Published:** 2019-02-15

**Authors:** Jimmy F. Zhang, Alex R. Paciorkowski, Paul A. Craig, Feng Cui

**Affiliations:** 1 Thomas H. Gosnell School of Life Sciences, Rochester Institute of Technology, One Lomb Memorial Drive, Rochester, NY 14623 USA; 2 Departments of Neurology, Pediatrics, Biomedical Genetics, and Neuroscience, University of Rochester Medical Center, 601 Elmwood Ave, Rochester, NY 14642 USA; 3 School of Chemistry and Materials Science, Rochester Institute of Technology, One Lomb Memorial Drive, Rochester, NY 14623 USA

**Keywords:** Virtual reality, GRIA2, User Interface, Data visualization

## Abstract

**Background:**

Functional characterization of single nucleotide variants (SNVs) involves two steps, the first step is to convert DNA to protein and the second step is to visualize protein sequences with their structures. As massively parallel sequencing has emerged as a leading technology in genomics, resulting in a significant increase in data volume, direct visualization of SNVs together with associated protein sequences/structures in a new user interface (UI) would be a more effective way to assess their potential effects on protein function.

**Results:**

We have developed BioVR, an easy-to-use interactive, virtual reality (VR)-assisted platform for integrated visual analysis of DNA/RNA/protein sequences and protein structures using Unity3D and the C# programming language. It utilizes the cutting-edge Oculus Rift, and Leap Motion hand detection, resulting in intuitive navigation and exploration of various types of biological data. Using *Gria2* and its associated gene product as an example, we present this proof-of-concept software to integrate protein and nucleic acid data. For any amino acid or nucleotide of interest in the *Gria2* sequence, it can be quickly linked to its corresponding location on Gria2 protein structure and visualized within VR.

**Conclusions:**

Using innovative 3D techniques, we provide a VR-based platform for visualization of DNA/RNA sequences and protein structures in aggregate, which can be extended to view omics data.

**Electronic supplementary material:**

The online version of this article (10.1186/s12859-019-2666-z) contains supplementary material, which is available to authorized users.

## Background

The advent of massively parallel sequencing (MPS) technologies in the past decade has revolutionized the field of genomics, enabling fast and cost-effective generation of a large amount of sequence data. This technological innovation leads to the accumulation of vast quantities of genomic data, posing a tremendous challenge to scientists for effective mining of data to explain a phenomenon of interest. To integrate the heterogeneous genomic datasets, genome viewers such as the UCSC Genome Browser [1], Ensembl [2], and the Integrative Genomics Viewer [3] adopt two-dimensional (2D) graph representations to display the data in compact and stacked tracks over genomic coordinates.

In the protein field, the number of protein sequences collected in public databases such as UniProt [4] has been growing exponentially over the last decade. The protein data bank (PDB) [2] holds more than 100,000 the atomic-level, three-dimensional (3D) protein structures. Both protein sequences and structures can be viewed by several tools including Cn3D [5], MultipSeq in VMD [6], STRAP [7], UCSF Chimera [8], and Aquaria [9]. These tools allow visualization of protein sequences and structures in separate panels of a 2D screen, which are manipulated by keyboard and mouse. However, none of these tools aggregate nucleic acid sequences with protein sequences and structures. As single-nucleotide variants (SNVs) are identified by massively parallel sequencing platforms, the successful aggregation of nucleic acid sequences can avoid the pre-processing step to translate SNV-containing mRNA sequences into protein sequences. Moreover, biological data across multiple domains should be visualized in the same environment, not in different panels.

Virtual reality (VR) provides a unique opportunity to address these challenges. VR refers to a 3D simulated environment generated by a computer into which users are immersed, as opposed to a 3D rendering of a 2D display [10]. In such an environment, users can observe internal complexity of the data, gain better understanding about the relationship among different elements, and identify previously unappreciated links between them. For these reasons, VR has potential benefits in abstract information visualization, and many studies have shown that users tend to understand data better and more quickly in VR environments than in conventional 2D and 3D desktop visualization [11–13].

Modern VR is based on a low-cost, stereoscopic head-mounted display (HMD), such as the Oculus Rift, Google Cardboard and HTC Vive, which presents different images to each eye to achieve 3D perception of the scene. In the case of Oculus Rift, HMD is accompanied by a head-tracking system using an infrared camera, a 3-axis gyroscope, and a hand-tracking device such as the Leap Motion. These developments in VR are very attractive to use in biological visualization because they give users intuitive control of exploring and manipulating complex biological data. Unsurprisingly, VR has been widely used for the visualization of biological data such as biomolecular [14] and metabolic [15] networks, microscopy images [16], protein-ligand complexes [17], biological electron-transfer dynamics [18], whole genome synteny [19], a whole cell [20], and medical education and research [21–28]. However, VR has not been used to integrate heterogeneous biomolecular sequence and structure data.

Here, we present a proof-of-concept application, BioVR, which is a VR-assisted platform to integrate and visualize DNA/RNA/protein sequences and protein structures in aggregate. The *Rattus norvegicus Gria2* gene (Glutamate Ionotropic Receptor AMPA type subunit 2) was used as a test case because its DNA sequence (on Chromosome 2, NC_005101), mRNA sequence (NM_001083811) and protein structure (5L1B) are well defined. This work allows researchers to visualize the DNA/RNA sequences of *Gria2*, together with its protein structures in VR.

BioVR provides an integrated view of nucleic acids, their protein products and the corresponding 3D structures. This is a new extension to the current tools that only display nucleic acid sequences (2D) or protein sequences (2D)/structures (3D). Our method can visualize all these biomolecules in the same environment (3D), enhancing our understanding of the sequence-structure relationship of SNVs. Because nowadays SNVs are usually identified by MPS technologies, our software could serve as a 3D genomic data viewer to visualize SNVs together with their protein sequences and structures.

## Implementation

### Hardware requirements

A VR-capable computer with the following specifications was used: (1) Intel Core i7-6700HQ Quad Core processor, (2) 6GB GDDR5 NVIDIA GeForce GTX 1060 graphics card, and (3) 8GB RAM. Oculus Rift with the following specifications was used: Oculus App (version 1.16.0.409144 (409268)) and device firmware (version 708/34a904e8da). To detect a user’s hands, Leap Motion Software (version: 3.2.0 + 45,899), Leap Motion Controller (ID: LP22965185382), and Firmware (version: 1.7.0) were used.

### Development tools

The VR-enabled desktop application was built using Unity3D (Unity), a game development environment with virtual reality capabilities (Additional file 1: Figure S1). It utilizes the C# programming language, which comes as part of Microsoft’s. Net software. Although both Unity and Unreal Engine support VR, Unity was chosen as the development platform for BioVR because it has ample online resources and is available on campus, through the virtual reality lab of the Center for Media, Arts, Games, Interaction and Creativity (MAGIC) of RIT.

A software package called UnityMol was created in Unity by Marc Baaden of Centre National de la Recherche Scientifique (CNRS). A non-VR 2014 version of this software, SweetUnityMol, is available for free download via SourceForge.net (Sweet UnityMol r676 beta r7) [29]. It is governed by a French copyright license called CeCILL-C which grants us the right to its free use and modification. With Unity version 5.4.2f, Sublime Text 3 Build 3126 (sublime), a code editor, and Git 2.11.0.windows.3 (git) for version control, we used the 2014 version (base code) as the basis of BioVR.

### Similarities and differences between BioVR and UnityMol

BioVR extends an open source 2014 version of UnityMol (https://sourceforge.net/p/unitymol/code-0/HEAD/tree/trunk/). In that particular codebase, the UnityMol project refers to itself by several version numbers. In the CHANGELOG it refers to itself as version 0.9.2, dated 2013-04-16. In the README, the version is marked as Revision 251. We refer UnityMol to this particular version 0.9.2.

Because BioVR extends UnityMol, the two codebases have a similar internal folder structure, with the major differences being that BioVR has additional directories for VR specific functionality. BioVR also adopts many of the software architecture conventions that UnityMol establishes, such as the Model-View-Controller architecture. Because UnityMol handles protein secondary structure rendering out of the box, BioVR also comes equipped with the ability to render protein secondary structure. Both projects are built with Unity, a software tool often used to develop computer games.

There are several major differences between BioVR and UnityMol. First, the two systems differ in how to implement the user interface (UI). Because UnityMol is not concerned with presenting proteins in VR, there is only one camera object through which the user views a protein. A VR environment requires a stereoscopic setting with two cameras offset by a small distance. To implement VR, we incorporated the Oculus SDK plugin into BioVR. The Oculus Software Development Kit (SDK) plugin feeds position and acceleration information from the Oculus headset to BioVR so that the stereoscopic cameras can mimic a user’s head movement.

Second, the two systems differ in how to control and interact with biomolecules. The mouse is the standard method of interaction in UnityMol. The user is expected to use the mouse to click on various panels and menus. In BioVR, we enable the use of hands to manipulate interface controls and to interact with the protein via Leap Motion and its associated SDK**.**

Third, the two systems differ in the integration of nucleotide sequence data. UnityMol is not built for the purpose of viewing nucleotide sequencing data. Rather, it specializes as a protein viewer and offers a variety of protein rendering options such as the ball and stick mode, secondary structure, or space-filling modes. BioVR de-emphasizes protein rendering and only offers the secondary structure rendering. Instead, it incorporates nucleotide sequencing data via a rectangular panel display that is situated close to “desk height.”

### Software design and development

#### Basic Unity objects

Software development within Unity constitutes its own specialty. A detailed discussion of Unity-specific challenges and best practices are beyond the scope of this paper. However, basic Unity concepts pertaining to BioVR are described below.

All objects in Unity are of type GameObject. A GameObject instance may contain zero or more components (Additional file 1: Table S1), including the MeshFilter. A MeshFilter object contains a reference to a Mesh instance. A Mesh instance can represent a single polyhedron by virtue of its internal data structures: vertices, triangles, normals, and UV coordinates (i.e.*,* coordinates scaled to the 0 .. 1 range for texturing a image, with 0 representing the bottom/left of the image and 1 representing the top/right of the image) arranged in arrays of the appropriate type, which has an upper vertex limit around 6000. In BioVR, the primary objects of interest are GameObject instances which contain a Mesh instance. A mesh can be accessed via the following:

$$ Mesh\ m= gameObject. getComponent< MeshFilter>\left(\right). mesh $$

assuming that there exists a non-null reference to GameObject named gameObject.

#### Game loop

The game loop is a ubiquitous concept in the gaming industry and influences BioVR in subtle but important ways. A game loop is a finite state machine that describes the high level game state at any given time point. It is modeled roughly as follows: when the player enters the game for the first time, the game loop starts. The current game state is rendered and displayed to the player. The player assesses the current game state and decides the next move, pressing the corresponding input(s). The next game state is computed based on the player’s inputs and scene information. As soon as the next game state is rendered on screen, it becomes the current game state. The player assesses the newly created game state and responds with inputs, repeating the loop. Unless game-ending conditions are triggered, the game loop continues.

Unity provides the MonoBehaviour class to help developers implement the game loop concept. The purpose of MonoBehaviour is to contain base code which organizes component actions into one of several game loop states. MonoBehaviour therefore contains the Awake(), Start(), and Update() functions, as well as other functions specific to Unity’s implementation of game loop design (Additional file 1: Table S2). To maintain game loop design principles, Unity encourages scripts to inherit from MonoBehaviour, but will otherwise compile and run normal C# classes with the caveat that those classes do not have the opportunity to directly affect game loop behavior.

**Fig. 1:**
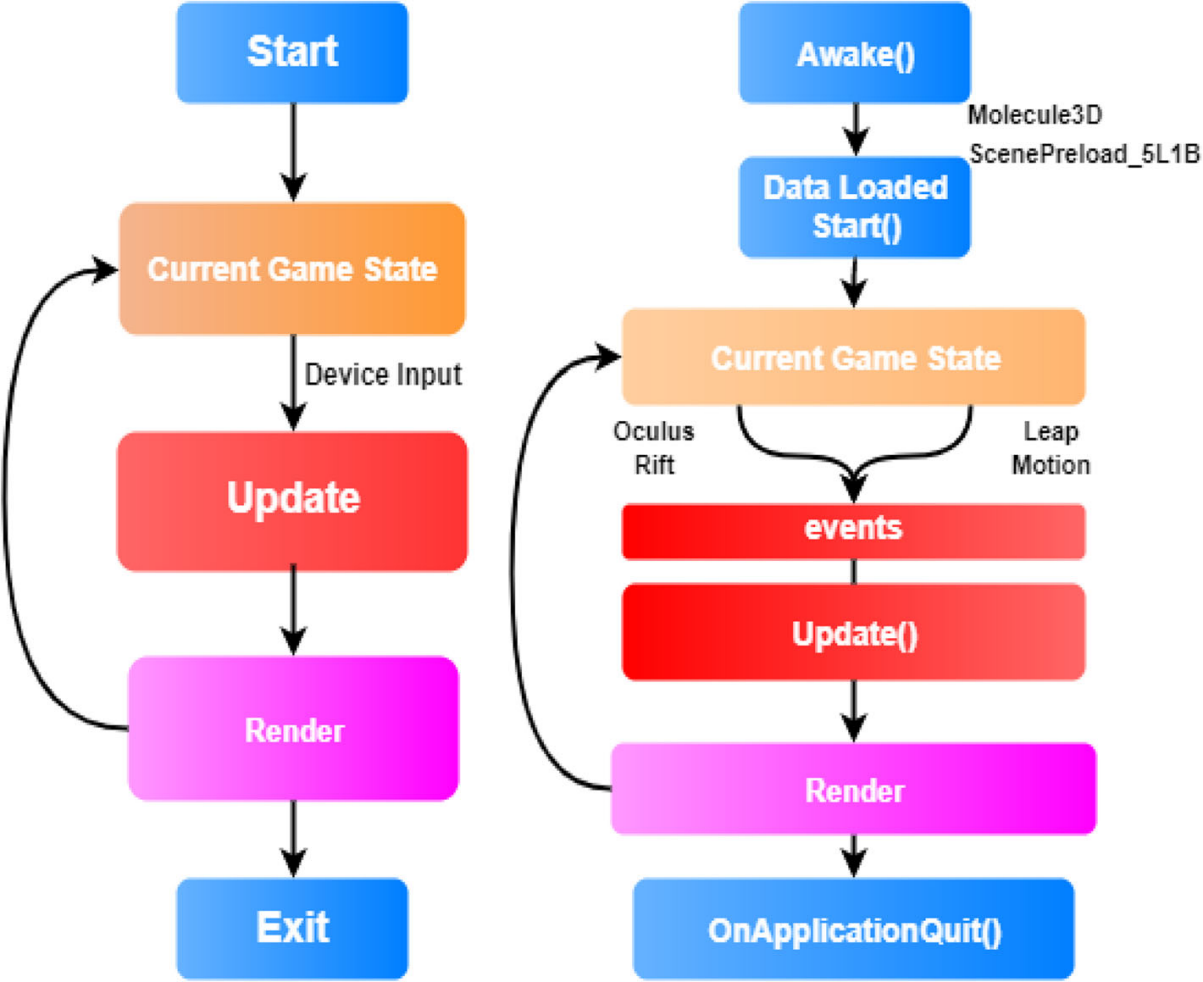
Comparison between the gaming operation and BioVR operation. (Left) A generic game loop. (Right) A high-level game loop specific to BioVR with important components that drive game state

VR applications rely on game loop architecture due to similarities in their state changes following user input. On start, the application takes on the state, *S0*, and information is rendered into the headset. Information about the player’s head orientation from the headset and finger positions from Leap Motion is gathered. The next state, *S*_*1*_, is then computed based on said information and rendered into the headset. Upon render completion, *S*_*1*_ becomes the current game state, and input from the headset and Leap Motion determines the next state *S*_*2*_. This continues to the state *S*_*N*_ so long as the user does not exit the application. The set of all states from *S*_*1*_ to *S*_*N*_ is part of the larger “playing” game state within the game loop. In BioVR, GameObjects that are subject to user input have some influence on the exact parameters of the next state and are therefore part of the game loop (Fig. 1). These GameObjects have attached components that inherit from MonoBehaviour and define the Awake(), Start(), and Update() functions as appropriate.

#### MVC

In UnityMol, the developers chose to implement the MVC architecture. BioVR is built on top of UnityMol and also adopts the MVC architecture. The Model View Controller (MVC) paradigm is a software architecture pattern commonly used to develop graphical user interfaces (GUIs). In a GUI, the software presents a visual representation of data to the user and awaits the user’s feedback. The user’s response is captured and induces some behind-the-scenes function, such as an update to a data model, or a query to a server. The response is captured by the data model and then presented to the user.

There are three main components in the MVC architecture—the model, the view, and the controller. The Model component is only responsible for representing data by storing it in sensible data structures and exposing appropriate functions to access all or parts of the data. The View component is primarily responsible for rendering the data. The View component has little influence over how the data is stored, so long as the Model component exposes a sensible application programming interface (API). The Controller mediates exchanges between Model and View components by responding to user input events. It is the sole determinant of what data subset is to be retrieved from the Model component and rendered via the View component, because it alone is “listening” to user input events.

### MVC implementation in UnityMol and BioVR

In UnityMol, there are separate namespaces for each MVC component and each namespace has its own folder. All of the components mentioned here are within the /Assets/Scripts subfolder unless explicitly stated otherwise.

The Molecule folder defines three subfolders corresponding to the three components of MVC. For example, in Molecule/Model/ there is a MoleculeModel.cs file which defines the molecule as a data model. In MoleculeModel.cs, the namespace Molecule.Model is defined: it contains the MoleculeModel class, which itself is a collection of data structures that collectively define the Molecule as a data model. The MoleculeModel file does not attempt to represent what a molecule is in chemistry or physics terms; it only defines such data structures that are useful to the UnityMol software. There are analogous examples for View and Controller components in the codebase (https://github.com/imyjimmy/gria2-viewer).

BioVR adopts the MVC convention set by UnityMol. There are several MVC components that are additions to UnityMol by virtue of the unique requirements of BioVR; these components are located in VRModel, View, and VRController folders.

### MVC model facilitates unique functionality

A model component that is particularly useful for visualization is the Residue.cs component, located in VRModel/. It works with other MVC components to bring about functionality unique to BioVR.

In UnityMol, the Splitting.cs file defines a Splitting class that helps to build the protein secondary structure for visualization. A List<Residue> represents the entire protein structure (in this case, the Gria2 structure) and is eventually passed into an instance of the Splitting class.

When a user hovers their hand over a position in the RNA Plane, the coordinate where the index finger touches is passed from an instance of RNAPanelController to a SequenceModel component that maps the coordinate to a particular residue. Each Residue object in List<Residue> contains a reference to the exact set of vertices that represents it in the secondary structure. Therefore, Unity’s shader is able to highlight the correct residue given the coordinate on the RNA Plane.

**Fig. 2:**
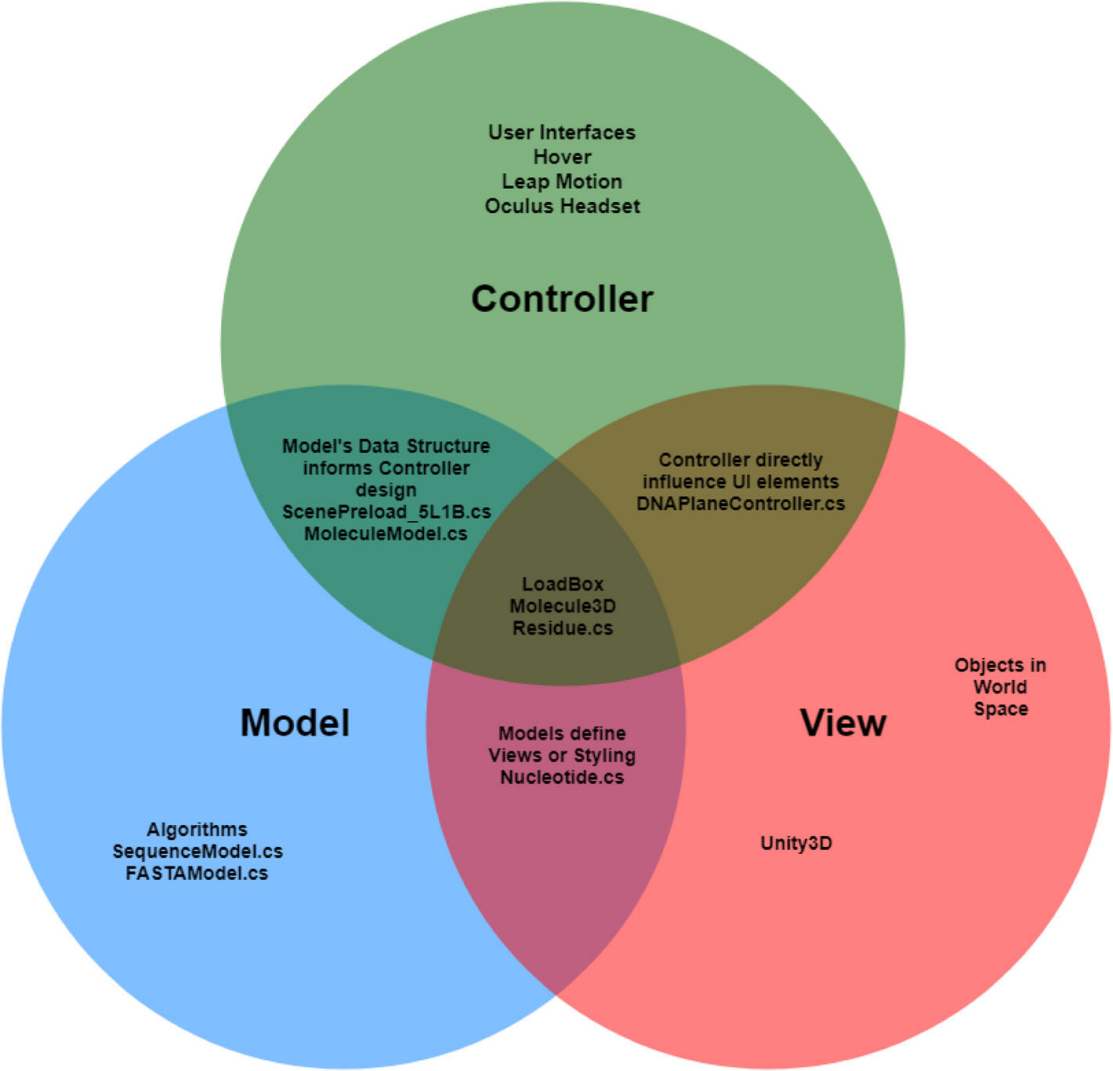
Major components of BioVR. Three separate namespaces including VRModel, View, and Controller were created to allow the Viewer faithfully execute on the MVC architecture. Note that certain components within the Viewer blur the line between different components of the ideal MVC model

Ideally, the three main components in the MVC architecture are well separated (Additional file 1: Figure S2). However, the MVC architecture in our codebase more closely resembles a Venn diagram (Fig. 2). We will improve it in our future release.

#### Data Models & Inheritance

**Fig. 3:**
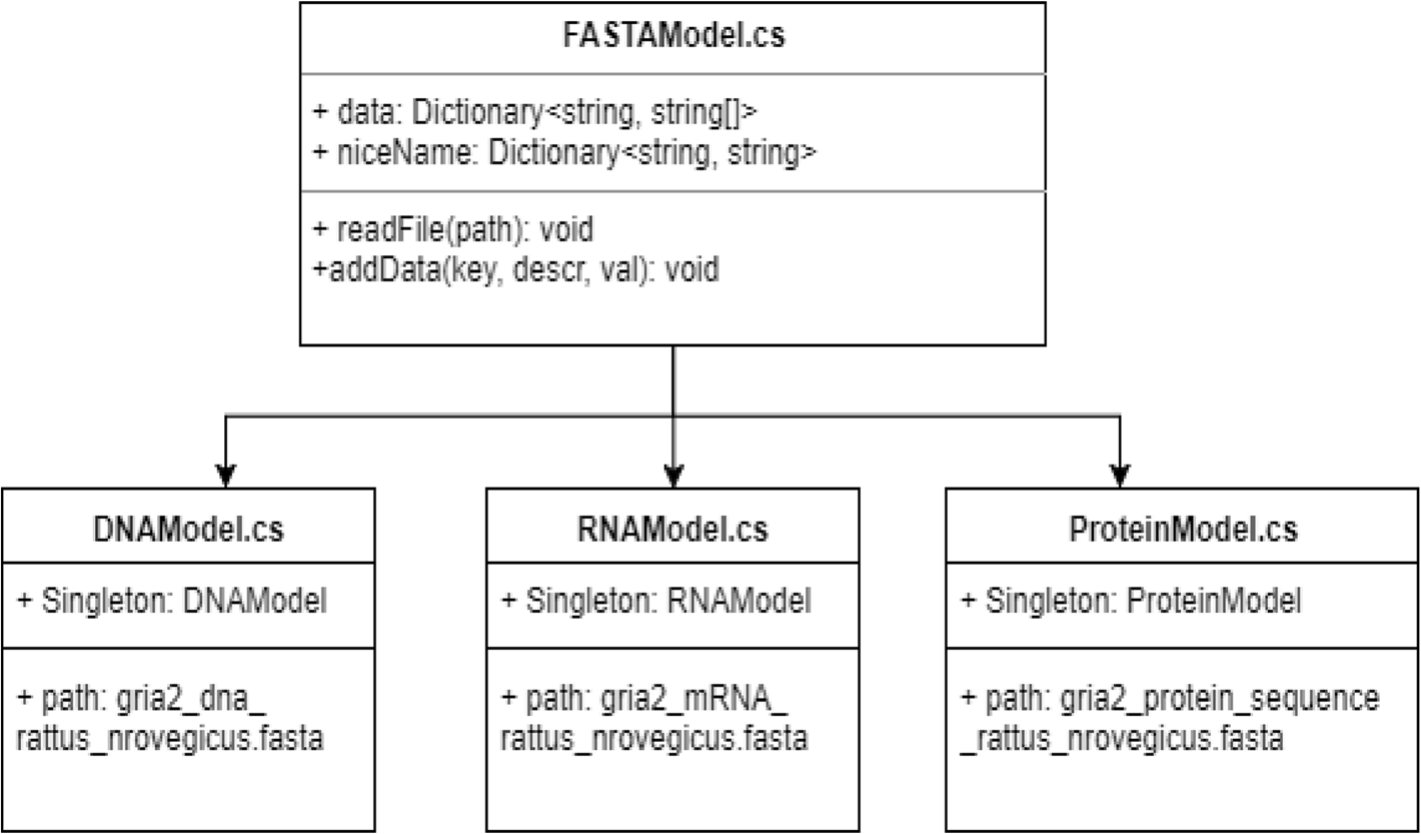
Inheritance relationship describing three different data models used in BioVR. The three data models, which are *DNAModel*, *RNAModel* and *ProteinModel*, are able to parse the data in the FASTA format. The *niceName* field maps common species names (e.g. *Homo sapiens*) to the key indices within the *data* field depending on the specific child class. The biological data of the *Gria2* gene (e.g., DNA, mRNA and protein sequences) were used for the purpose of illustration

The advantage of inheritance in software development is twofold: it promotes code reuse by forcing the developers to assess the common features of particular code modules, and it groups assumptions about modules into closely related and easy-to-find packages. (https://www.cse.msu.edu/~cse870/Input/SS2002/MiniProject/Sources/DRC.pdf) In BioVR, object-oriented inheritance is utilized only to describe data models for parsing and holding biological data files (Fig. 3).

**Fig. 4:**
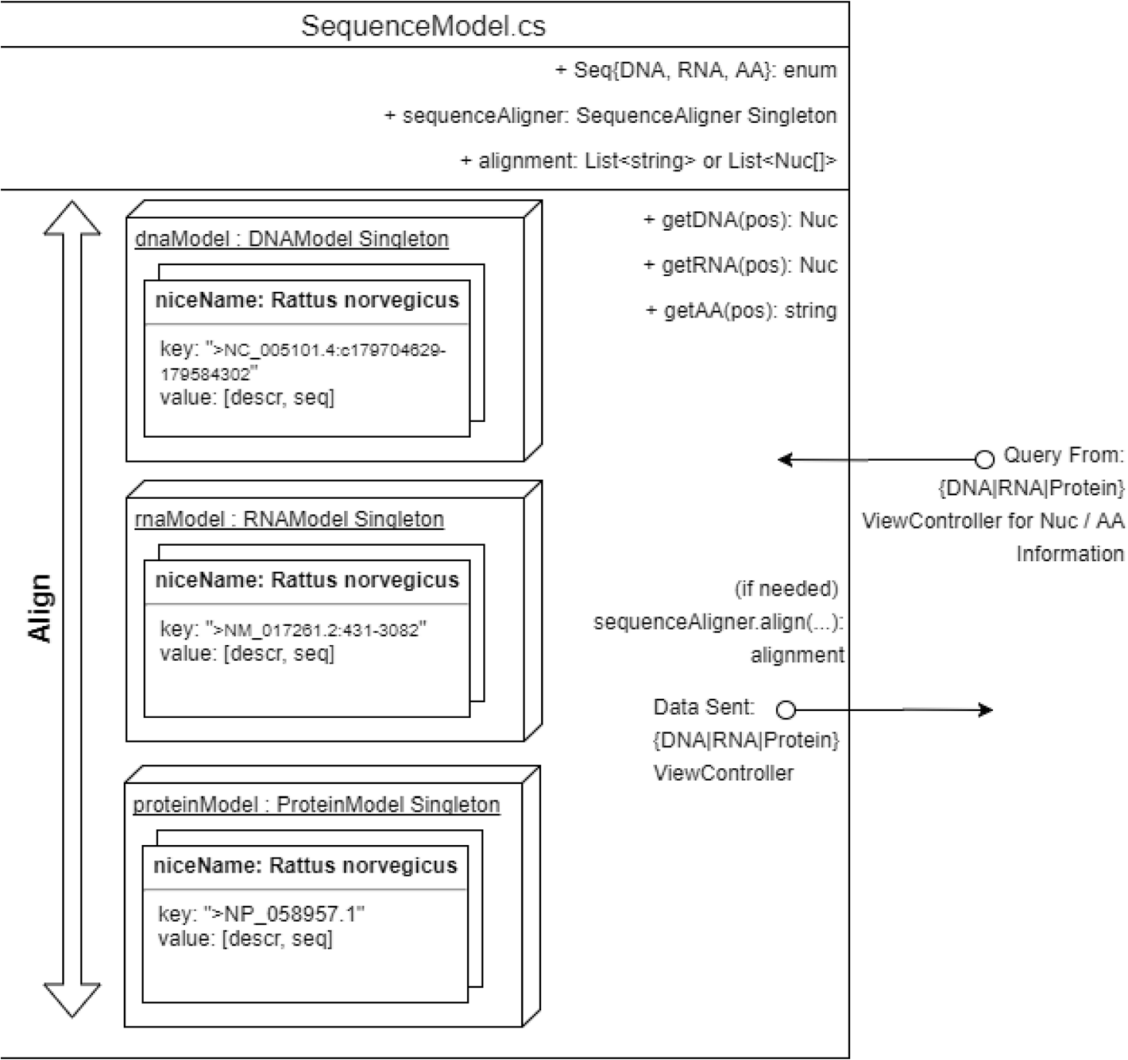
Implementation of the sequence model *SequenceModel.* The model keeps references to DNAModel, RNAModel, ProteinSeqModel singletons, and a reference to a SequenceAligner instance that can implement the Needleman-Wunsch algorithm for global sequence alignment

All FASTA files are processed by the same way; therefore the common parsing code resides within the *FASTAModel.cs* module. Each species for which FASTA files are available map to a unique *niceName* string instance, e.g. string *niceName* = “*Rattus norvegicus*”. The *niceName* string instance is common to the DNA, RNA, and Protein models for each species, but they map to different valued keys within the data dictionary, depending upon which type of model (DNA, RNA, or Protein) that the user is interested (Fig. 4).

#### UI

Hover UI Kit is a free software package available for download from github. It is governed by a GPLv3 license which authorizes free use for open source projects. Once Hover UI libraries are imported into the project directory, an empty GameObject is created, and a Hover UI creation script component is attached. Running the script directly in Unity editor mode results in a static menu set, if given appropriate parameters. The static menu instance is directed to find and attach itself to an instance of Leap Motion hands at runtime (Additional file 1: Figure S3). The left-hand transform acts as the parent to the UI menu while the right hand acts as a pointer. The complete menu hierarchy as implemented in BioVR is listed in Additional file 1: Table S3.

#### Plane geometry and UV coordinates

We use plane geometry and UV coordinates to map nucleotide sequences onto UI elements. A Unity plane geometry is a flat surface as defined by variables contained in its mesh instance. Every mesh instance has an array of Vector2 UV coordinates (Fig. 2) which define UV the mapping of a two-dimensional texture image onto the projected surface of any valid geometry. In the case of plane geometry, UV coordinates map perfectly to the length and width of the plane such that in the default case, the U coordinate ranges from (0, 1) and spans the plane’s length, whereas the V coordinate ranges from (0, 1) and spans the plane’s width.

To render nucleotide sequences onto textures, we take advantage of two properties. First, geometries have the unique property of being allowed to partially map to UV coordinates. In other words, mesh geometries do not need to span the entire UV range. Second, Unity allows the procedural editing of textures via *Texture2d.SetPixel*(int x, int y, Color color) where x, y refers to a texture coordinate. Note that a 256 × 256 texture will map to a 1 × 1 UV square such that (0,0) = > (0,0) and (256, 256) = > (1,1). Thus, each nucleotide within a sequence can be represented by their traditional colors (Additional file 1: Table S4) and associated with a specific (u, v) coordinate.

Since geometries don’t need to map to the entire UV space, the *MeshRenderer* component of the plane geometry mesh will then only render portions of the nucleotide sequence at a time (Additional file 1: Figure S4). The range of the nucleotide sequence to be rendered can be adjusted via scrolling.

### Test datasets

We used the sequences and structures of glutamate ionotropic receptor AMPA type subunit 2 (Gria2) as the test dataset of BioVR as tertiary structure of this protein is available to accompany the genetic sequence. This subunit is one of the four Gria subunits (Gria1–4) involving in the assembly of cation channels (glutamate receptors) activated by alpha-amino-3-hydroxy-5-methyl-4-isoxazole propionate (AMPA). Glutamate receptors play a vital role in mediating excitatory synaptic transmission in mammalian brain and are activated in a variety of normal neurophysiologic processes. Prior studies have identified glutamate binding and closing mechanisms using PDB: 1FTO [30] with the caveat that 1FTO only captures the ligand binding core of the protein. Of the five structures submitted to PDB, 5L1B shows the Gria2 structure in the apo state; therefore 5L1B was selected for use in this project due to its symmetry and unbound nature. Source files containing the Gria2 structure (5L1B) were downloaded from Protein Data Bank (PDB).

Both sequence and structure data of Gria2 from *Rattus norvegicus* are preloaded into the StreamingAssets folder of the BioVR project. BioVR builds have access to a compressed version of the StreamingAssets folder at runtime. During the User Interface Case Study, we plan to provide the equivalent of “built-in” access to these documents for subjects randomized to the Traditional Computer group. A Chrome browser will be open with tabs corresponding to the NCBI page for these documents.

## Results

### Gria2 in BioVR: A test case

**Fig. 5:**
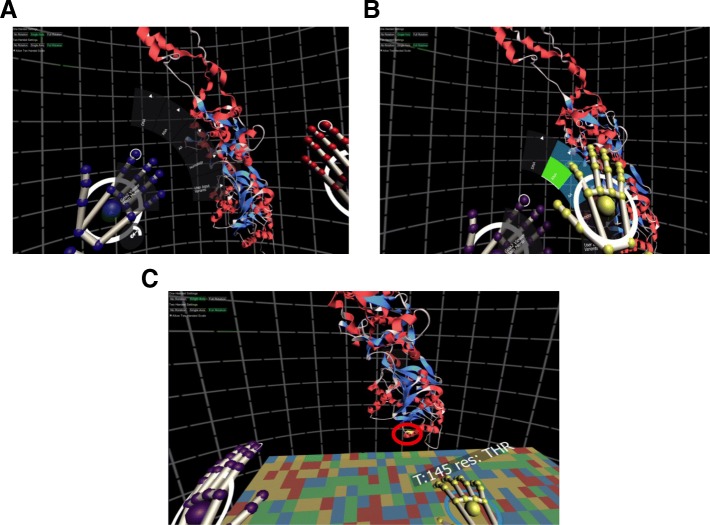
Direct visualization of the nucleotide sequence and protein structure of rat Gria2 in BioVR. **a** Navigation within BioVR. BioVR uses a menu set anchored to the user’s left hand. The menu set is an instance of Hover UI, an open source project found at https://github.com/aestheticinteractive/Hover-UI-Kit. **b** Hover UI used in conjunction with Leap Motion. The menu is anchored to the user’s left hand. The index finger on the right hand acts as a cursor: it generates a button pressed event for a particular button when it hovers over that button within a set amount of time. The specific timing varies and can be set per button. **c** *Rattus norvegicus Gria2* RNA sequence and protein structure shown in BioVR. The mRNA sequence of rat Gria2 (NM_001083811) was loaded in the RNA Panel. Above the right index finger is a GUI which displays context-sensitive data depending on where the user places his or her index finger along the sequence. The residue corresponding to the mRNA nucleotide is also highlighted in the 3D structure (PDB ID: 5L1B) via a yellow outline shader (denoted by red circle). Note that the red circle was added for the illustration purpose and does not appear in the actual program

BioVR allows scientists to view, at the same time, DNA, RNA and protein (called AA in UI) data. In our test case we visualized the *Gria2* gene in a VR-assisted visualization platform. It features a simple user interface for viewing genomic information which utilizes Leap Motion tracking to turn fingers into data manipulators. To the right hand is attached an instance of Hover UI. The user can select to view DNA, RNA or protein sequences (Fig. 5a-b).

### Nucleotide sequences and UV coordinates

The ideal representation of modestly large (~ 100 Kb) nucleotide sequences is an open problem in both VR as well as web interfaces. We created a plane geometry with customized UV coordinates that allow for the representation of nucleotide sequences of up to 100 Kb (Additional file 1: Figure S4). Using the *BuildTexture()* method in {DNA | RNA}PanelController, the appropriate FASTA file is accessed and its nucleotide sequence is processed such that each texture coordinate of the DNA or RNA plane takes on a color that represents a specific nucleotide in the FASTA sequence. The protein sequence in one letter code can be overlaid on top of the mRNA panel (Fig. 5c). If the user selects “AA>Show on Model” and goes to “RNA > Show”, BioVR gives additional context. Specifically, it displays the 3D structure together with the mRNA sequence of Gria2; the position of selected nucleotide and its corresponding residue on the 3D structure are shown (Fig. 5c).

## Discussion

Milestones within the technology industry are often marked by major UI innovations. The shift in UI, for example, from the mouse in Windows to touchscreen, has a tangible influence on efficiency and profound impact on user growth and consumer adoption. Similarly, studies have shown that researchers tend to understand data more quickly in VR environments than in conventional 2D and 3D desktop visualization [11–13].

In this paper, we present BioVR, an easy-to-use, interactive VR-based platform, to aggregate sequence and structure data of nucleic acids and proteins. This tool aims to fill two knowledge gaps in bioinformatics: the first is in SNV analysis by integrating nucleic acid sequences with protein sequences/structures, and the second is in VR visualization technology by applying VR to biomolecular data. As a result, users can directly assess the effects of identified SNVs by mapping the corresponding residues on protein structures. This will avoid a time-consuming pre-processing step in which users need to switch different tools back and forth to gather information they need. Meanwhile, BioVR displays all data in 3D, and this will avoid the switching between the structure panel (3D) and the sequence panel (2D) used in current tools.

We have developed the following strategy to compare the efficiency of using BioVR versus currently available tools such as Aquaria to find SNVs of interest and locate the corresponding residues on protein structure. Use the *Gria2* gene as an example. There are 6 SNVs in this gene. Ten junior or senior bioinformatics students will find these SNVs from NCBI databases and map corresponding residues on Gria2 structure. The time students need to accomplish the task using BioVR or Aquaria will be recorded, compared and analyzed. This test will be repeated for 10 genes/proteins in which both sequence and structure data are available, and the results will be published elsewhere.

As mentioned above, BioVR is a proof-of-concept software, which has several limitations and needs further improvement. First, BioVR is currently limited to displaying data for a single gene (i.e., the *Gria2* gene). There are plans to enable the display of proteome and genome data for other loci. Once a database of proteins on PDB and their genomic counterparts are correctly matched, BioVR can trivially load VR representations of other loci. Second, the DNA Panel supports scrolling, but the popups that are triggered when the user moves their fingers over the panel are not accurate if scrolling has been engaged. Third, BioVR does not support a visual analysis of sequence alignments. The ability to visually report sequence similarity is a planned feature. Currently, the DNA panel reports the nucleic acid of a particular sequence position via color. For a future release, we anticipate that the DNA panel can also represent the results of alignment algorithms by adjusting the transparency or brightness of each position. The alignment algorithms that support this have not been natively implemented.

Recent development in genomics and epigenomics has accumulated a large amount of data. These data are currently visualized in track-based genome browser such as UCSC’s Genome Browser [1], Ensembl Project [2], and Integrative Genomics Viewer (IGV) [3]. BioVR can be extended to view these data in the 3D representation of a genome whose conformations are defined by chromosome conformation capture techniques such as Hi-C.

## Conclusions

Virtual reality is a ground-breaking medium with major advantages over traditional visualization for biological datasets. Its potential remains largely unexplored. Here, we develop an easy-to-use, VR-assisted platform, BioVR, and show that DNA/RNA sequences and protein structures can be viewed in aggregate, leading to a novel workflow for researchers.

Our work can be extended to view MPS data to assess the effects of SNVs on protein function. Whole genome representation can be rendered at low resolution until users decide to investigate a gene locus of interest, upon which the VR application may zoom in to the region at higher resolution. Interactions among promoters, enhancers, and silencers at the loci can be shown depending on chromatin context. Geometric topologies within chromatin regions will be made obvious to the user. Finally, animated simulations can be made to help users visualize temporal datasets.

## Availability and requirements

**Project name:** BioVR.

**Project home page**: https://github.com/imyjimmy/gria2-viewer

**Operating system:** Windows 10.

**Programming languages**: C#.

**Other requirements**: Unity 5.4.2f, Leap Motion, Oculus SDK 1.11.0, Hover UI Kit 2.0.0 beta.

**License:** GNU General Public License.

## Additional file

Additional file 1: BioVR: a platform for virtual reality assisted biological data integration and visualization. Contains additional figures and tables of the research. (PDF 974 kb)

## Abbreviations

*Gria2*: Glutamate Ionotropic Receptor AMPA type subunit 2
HMD: Head-mounted display
MPS: Massively parallel sequencing
MVC: Model-view-controller
SoC: Separation of concerns
UI: User interface
