# Supplementary material for: BioVR: a platform for virtual reality assisted biological data integration and visualization
Source: BMC Bioinformatics. 2019 Feb 15;20:78. doi: 10.1186/s12859-019-2666-z (PMC6376704; doi:10.1186/s12859-019-2666-z)
Supplement: Supplementary file 1 — BioVR: a platform for virtual reality assisted biological data integration and visualization. Contains additional figures and tables of the research. (PDF 974 kb) [file 12859_2019_2666_MOESM1_ESM.pdf]

Supplementary Materials

BioVR: a platform for virtual reality assisted biological data integration and visualization

Jimmy F. Zhang[1*], Alex R. Paciorkowski[3], Paul A. Craig[2], and Feng Cui[1*]

[1]Thomas H. Gosnell School of Life Sciences, [2]School of Chemistry and Materials Science, Rochester Institute of Technology, One Lomb Memorial Drive, Rochester, NY 14623, USA [3]Departments of Neurology, Pediatrics, Biomedical Genetics, and Neuroscience, University of Rochester Medical Center, 601 Elmwood Ave, Rochester, NY 14642, USA

Contents

Supplementary Figures 2

Figure S1 2
Figure S2 3
Figure S3 4
Figure S4 5

Supplementary Tables. 6

Table S1 6
Table S2 7
Table S3 8
Table S4 9

Supplementary Methods 10

Installation Instructions. 10
System Requirements & Installation. 10
Installation Instructions. 11
Code Excerpts. 12
Nucleotide.cs. 13
Residue.cs. 13
FASTAModel.cs. 13

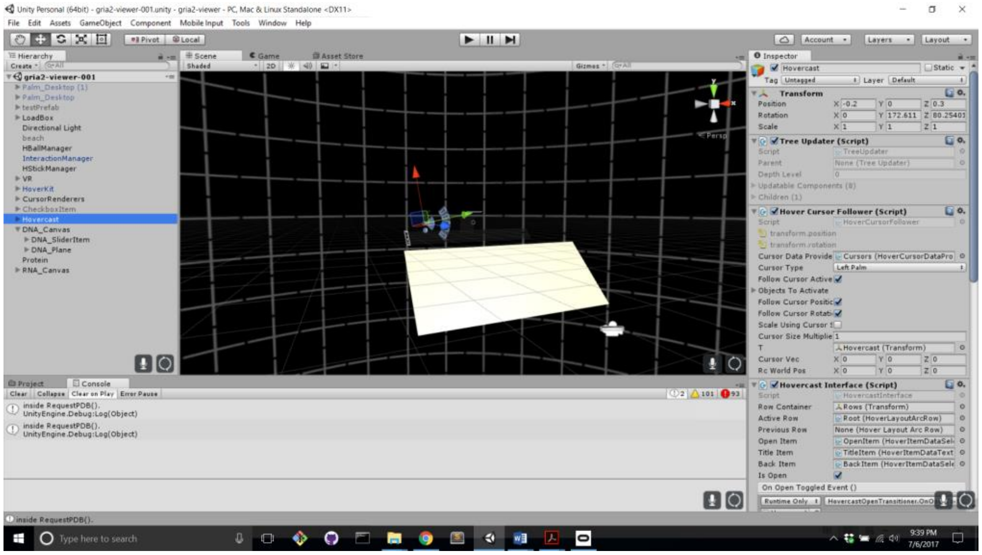

Figure S1. Unity’s user interface.

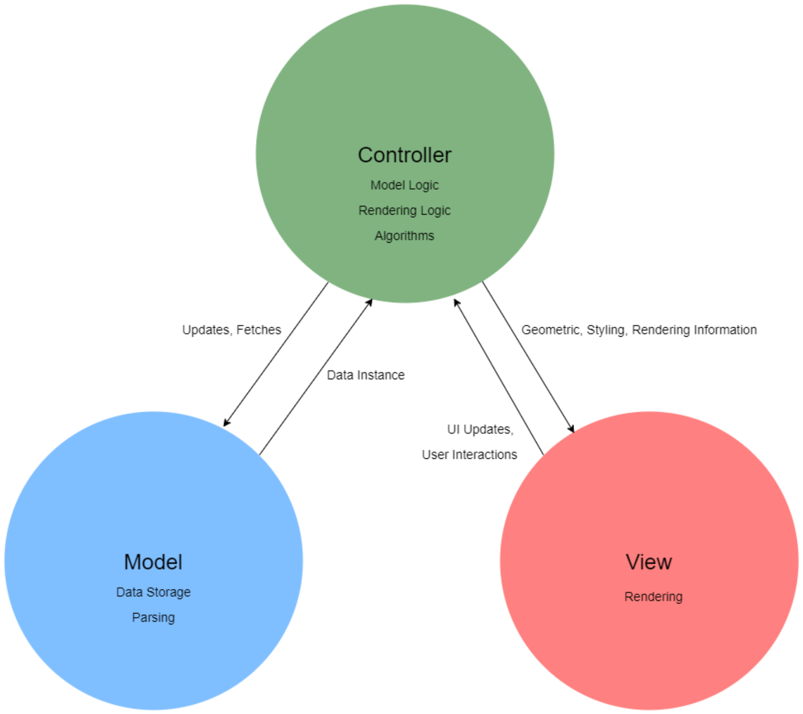

Figure S2. The ideal MVC architecture involves complete separation of concerns.

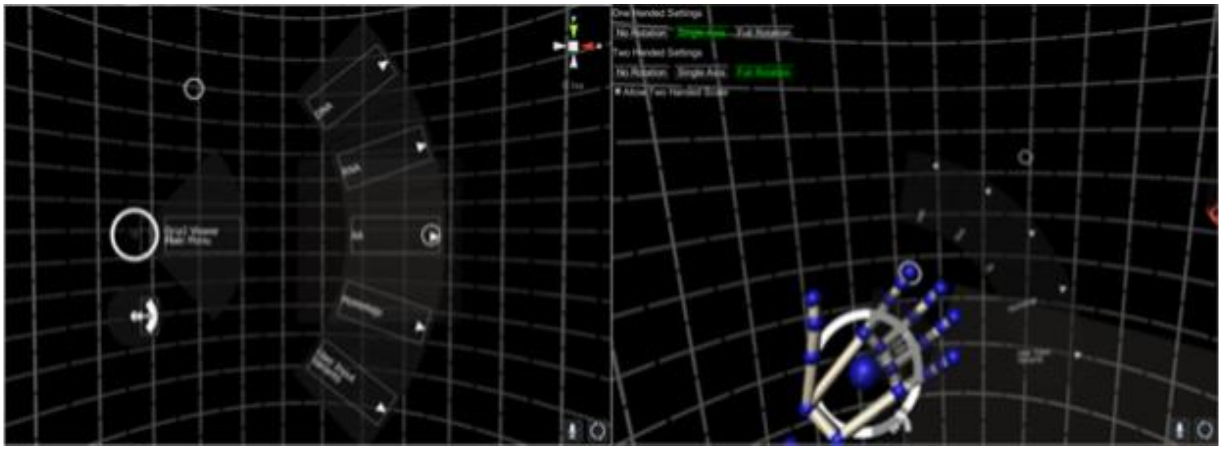

Figure S3. Hover UI v 2.0.0-alpha used in conjunction with Leap Motion Orion v3.0.0. The menu is anchored to the user’s left hand. The index finger on the right hand acts as a cursor: it generates a button pressed event for a particular button when it hovers over that button within a set amount of time. The specific timing varies and can be set per button.

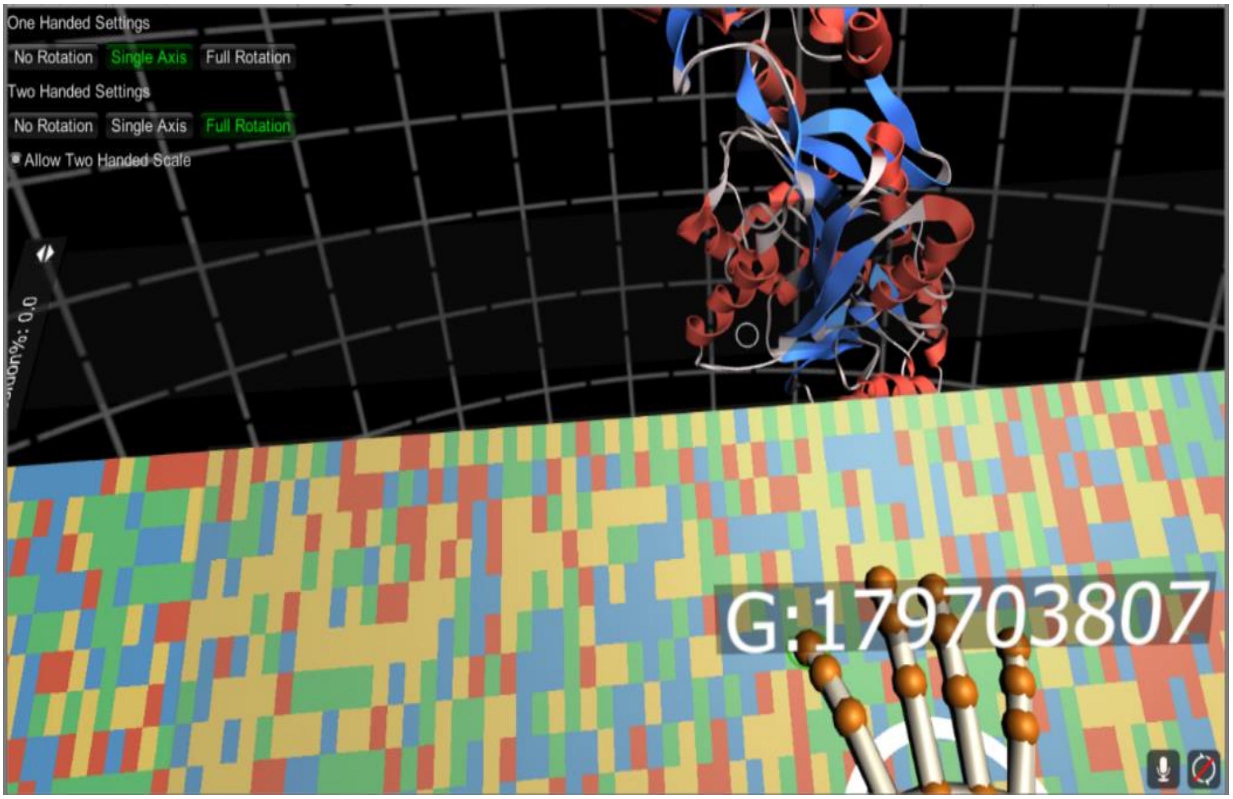

Figure S4. *Rattus norvegicus Gria2* DNA sequence loaded into the DNA Panel. The *Gria2* DNA sequence (120,327 bp) was given in a FASTA format in which the start and end positions of the sequence on Chromosome 2 (NC_005101) are provided in the header. As the user's right index finger hovers over a point on the panel, the nucleotide for that point is retrieved; it is displayed along with the position in rat Chromosome 2 at which the nucleotide is located.

Supplementary Table S1. Common objects in Unity and their uses.

Class	Description	Contains	Parent in Scene Hierarchy	Parent in Object Oriented Inheritance
**GameObject**	Base class for all entities in Unity scenes.	Empty, Transform Component, other Components, or other GameObjects.	Other GameObjects, Unity scene	Object
**Component**	Base class for everything attached to GameObjects.	A reference to the GameObject to which the component is attached.	Is attached to a GameObject but has no parent since it isn't represented in scene hierarchy.	Object
**MeshFilter**	Accessor class for Mesh objects. Passing Mesh info to MeshRenderer for rendering.	Mesh object.	Is attached to a GameObject, but has no parent for the same reason as Component.	Component
**MeshRenderer**	The Mesh Renderer takes the geometry from the Mesh Filter and renders it at the position defined by the object's Transform component."	References to Lighting, Material objects used to render geometry.	Is attached to a GameObject, but has no parent for the same reason as above.	Renderer > Component
**Mesh**	Represents a geometry, e.g. a plane, cube, or any other polyhedron.	int[] triangle, Vector3[] vertices, Vector3[] normals,	Retreived via MeshFilter.	Object
**MonoBehaviour**	Base class from which all Unity scripts derive.	Awake(), Start(), Update() functions	Scripts which inherit MonoBehaviour are attached to GameObject instances.	Behaviour > Component

Note: Instances of MeshFilter, MeshRenderer, and other classes are often refered to as a type of Component when attached to a GameObject.

Supplemental Table S2. A summary of MonoBehaviour functions for enforcing game states. These functions are helpful for maintaining the application state within the game loop.

**public Monobehaviour functions**	**Call Frequency & Timing**	**Role**
`Awake()`	Once, when the script instance is being loaded.	"Used to initialize any variables or game state before the game starts."
`Start()`	Once, when the script is enabled, before any Update() functions are called	Facilitates interactions between enabled components. Other components may not be enabled during Awake().
`Update()`	Every frame, after Start() is called	Implements change in state / behavior
`FixedUpdate()`	At certain framerate intervals, eg. at frames 24, 48, 72, etc.	Computationally expensive state changes
`LateUpdate()`	Every frame, after all Update() functions are called.	Ordering events
`OnGUI()`	Whenever a GUI event is fired	Handling GUI events
`OnDisable()`	Called when component is disabled or becomes inactive	Teardown code and associated routines
`OnEnabled()`	Called when component is enabled or becomes active	Component initialization code

Supplementary Table S3. The complete hierarchical menu set is an instance of Hover UI, and open source project for VR interfaces.

- Gria2-Viewer Main Menu
  - DNA
    - Show
    - *Coding Region (CDS)*
    - Back
  - RNA
    - Show
    - *Consensus (MSA)*
    - Back
  - AA
    - Show on Model
    - *Consensus*
    - Back
  - Homology
    - Rattus norvegicus
    - *Homo sapiens*
    - *Pan troglodytes*
    - Back
  - User Input Variants
    - *Variant 1*
    - *Variant 2*
    - *Variant 3*

Note: Italics represent buttons which will be implemented in a future release.

Supplementary Table S4. Nucleotides in DNA and RNA Panels are represented by the colors listed above.

Nucleotide	Color	RGB
**A**	Blue	68,155,255
**T / U**	Yellow	244,220,110
**C**	Red	224,81,62
**G**	Green	83,209,131

Note: Each pixel can be set to a unique color using the Texture2d.SetPixel function. Thus, a sequence of *n* nucleotides will generate *n / textureWidth* rows of *textureWidth* color squares. Each color square represents the nucleotide at a unique sequence position.

Excerpts from Documentation

The following is an excerpt of installation instructions from the documentation for Gria2-Viewer. The complete documentation can be found at https://github.com/imyjimmy/gria2-viewer.

System Requirements & Installation

Hardware

Minimum Specs

- VR-Capable PC
    - Graphics Card: NVIDIA GTX 1050 Ti / AMD Radeon RX 470 or greater
    - Alternative Graphics Card: NVIDIA GTX 960 4GB / AMD Radeon R9 290 or greater
    - CPU: Intel i3-6100 / AMD FX4350 or greater
    - Memory: 8GB+ RAM
    - Video Output: Compatible HDMI 1.3 video output
    - USB Ports: 1x USB 3.0 port, plus 2x USB 2.0 ports
- Oculus Rift CV1 (DK2 not tested)
- Leap Motion Controller

Recommended PC Specs

- Graphics Card: NVIDIA GTX 1060 / AMD Radeon RX 480 or greater
- Alternative Graphics Card: NVIDIA GTX 970 / AMD Radeon R9 290 or greater
- CPU: Intel i5-4590 equivalent or greater
- Memory: 8GB+ RAM
- Video Output: Compatible HDMI 1.3 video output
- USB Ports: 3x USB 3.0 ports, plus 1x USB 2.0 port
- OS: Windows 10

Exact PC Specs Used

The Gria2-Viewer project is tested on the following hardware:

- Eluktronics P650RP6 Premium VR Ready Gaming Laptop
- Graphics Card: 6GB GDDR5 NVIDIA GeForce GTX 1060
- CPU: Intel Core i7-6700HQ Quad Core
- Memory: 8GB DDR4 RAM
- Storage: 128GB Performance SSD + 1TB HDD
- OS: Windows 10 Home

- Full HD 15.6" IPS

*Software**

- OS: Windows 8.1 or newer
- Unity 5.4.2f
- Leap Motion Software Modules:
  - SDK: 3.2.0+45899 (Orion) Download Here
  - Core Assets: 4.1.5 Download Here
  - Interaction Engine v0.3.0 Download Here
  - Hands Module 2.1.0 Download Here
- Oculus SDK 1.11.0 Download Here
- Hover UI Kit 2.0.0 beta Download Here

***Software must be exact versions!!**
Other software includes: git and your favorite text editor.

Installation Instructions

Components should be installed in the following order:

1. Unity
2. Plug in and install Oculus device
3. Oculus SDK
4. Attach Leap Motion sensor to Oculus Headset; install Leap Motion Software
5. Download Gria2-Viewer from github here and open `Assets/gria2-viewer-001.unity` with Unity
6. Leap Motion SDK
7. Leap Motion Core Assets
8. Leap Motion Hands Module
9. Leap Motion Interaction Engine
10. Hover UI Kit

At each of these installations it is **highly recommended** that you make a bare-bones Unity project to test that the components have been added. Use the provided instructions from each of the software vendors. Requires working knowledge of git and preferably bash for Windows.

Code Excerpts

Nucleotide.cs

```
/*
* @imyjimmy
*/
namespace VRModel.Monomer {
   using UnityEngine;
      using System;
      using System.Collections;
      using System.Collections.Generic;

      public enum Nuc {A,T,C,G,X}; //X is used to mark where MSA
alignment skips.

      public class Nucleotide {

            public static readonly Dictionary<Nuc, string> nucStr = new
Dictionary<Nuc, string> {
                  {Nuc.A, "A"},
                  {Nuc.T, "T"},
                  {Nuc.C, "C"},
                  {Nuc.G, "G"},
                  {Nuc.X, "-"}
            };

            public static readonly Dictionary<string, Nuc> strNuc = new
Dictionary<string, Nuc> {
                  {"A", Nuc.A},
                  {"T", Nuc.T},
                  {"C", Nuc.C},
                  {"G", Nuc.G},
                  {"-", Nuc.X}
            };

            public static readonly Dictionary<Nuc, Color32>
defaultColor = new Dictionary<Nuc, Color32> {
                  {Nuc.A, new Color32(68,155,255,255)},
                  {Nuc.C, new Color32(224,81,62,255)},
                  {Nuc.T, new Color32(244,220,110,255)},
                  {Nuc.G, new Color32(83,209,131,255)},
                  {Nuc.X, new Color32(99,99,99,255)}
            };

            public static Nuc charToNuc(char c) {
                  return strToNuc(Char.ToString(c));
            }

            public static Nuc strToNuc(string x) {
                  try {
```

```
                        return (Nuc) Enum.Parse(typeof(Nuc), x);
                    }
                    catch {
                        throw new Exception();
                    }
                }

                public static string NucToStr(Nuc n) {
                    return n.ToString();
                }
            }
        }
```
Code Excerpt 1: Nucleotide.cs defines nucleotide enums, their color schemes under certain conditions, and the mappings between strings and enums. The namespace VRModel was used due to a conflict with an existing namespace Model.

Residue.cs

```
//@imyjimmy
namespace VRModel {
	using System.Collections;
	using System.Collections.Generic;
	using UnityEngine;

	public class Residue {
		public string name { get; set; }

		public List<int> triangles { get; set; }
		public List<Color32> colors { get; set; }
		public List<int> vertices { get; set; }
		public List<int> normals { get; set; }

		//if Splitting.cs separates the protein into more than 1 
mesh,
		//the mesh number list refers to the mesh numbers in which 
the residue is represented.
		//colors, verts, normals and triangle indices are all reset 
with respect to the current mesh.
		//(I think)
		public List<int> meshIndices { get; set; }
	}
}
```

Code Excerpt 2: PDB data is parsed and converted to meshes called Ribbons. Residue.cs keeps track of which vertices, triangles, colors and normal belong to a given amino acid. A list of type List<Residue> allows for the mapping of residue sequence information to the specific set of vertices that represent the given residue. Due to vertex limits, some amino acids may span multiple geometries (Ribbons). List<int> meshIndices lists all of the indices corresponding to the List<mesh> index in which they can be found. Most Residue instances list one int in meshIndices because splitting the ribbon geometry into two only occurs when the upper vertex limit for a particular mesh is reached.

FASTAModel.cs

```
//author: @imyjimmy
//loads fasta files.

namespace VRModel.Monomer {
	using UnityEngine;
	using System.Collections;
	using System.Collections.Generic;
	using System.IO;
	using System.Text.RegularExpressions;
	// using UI;

	public class FASTAModel {

		public Dictionary<string, string[]> data; //key:
">NM_001001775.3"
									//note: DNAModel key
contains: complement, indexStart, indexEnd, etc.
									//value : [descr, sequence]
									//@todo: [descr,
indexStart, indexEnd, sequence]
									//value example: ["Gallus
gallus glutamate ionot...subunit 2 (GRIA2),...mRNA",
									// ATTATCCC...]
		//public string niceName;
		public Dictionary<string, string> niceName;

		public FASTAModel() {
			data = new Dictionary<string, string[]>();
			niceName = new Dictionary<string, string>();
		}
...
```

Code Excerpt 3: FASTAModel.cs parses FASTA files and distributes their data among the data Dictionary instance. An example entry in data would be: { “>NM_001001775.3” => [“Gallus gallus glutamate ionotropic receptor subunit 2 (GRIA2), mRNA”, “ATTATCCAT…”]}
